# A crystallographic perspective on sharing data and knowledge

**DOI:** 10.1007/s10822-014-9780-9

**Published:** 2014-08-05

**Authors:** Ian J. Bruno, Colin R. Groom

**Affiliations:** The Cambridge Crystallographic Data Centre, 12 Union Road, Cambridge, CB2 1EZ UK

**Keywords:** Crystallography, Data, Knowledge, Sharing, Sustainability

## Abstract

The crystallographic community is in many ways an exemplar of the benefits and practices of sharing data. Since the inception of the technique, virtually every published crystal structure has been made available to others. This has been achieved through the establishment of several specialist data centres, including the Cambridge Crystallographic Data Centre, which produces the Cambridge Structural Database. Containing curated structures of small organic molecules, some containing a metal, the database has been produced for almost 50 years. This has required the development of complex informatics tools and an environment allowing expert human curation. As importantly, a financial model has evolved which has, to date, ensured the sustainability of the resource. However, the opportunities afforded by technological changes and changing attitudes to sharing data make it an opportune moment to review current practices.

## Introduction

Over half a century ago, crystallographers decided to make crystal structure data available in a systematic way. Motivated by Bernal [1], the reasons behind this were later expressed rather eloquently by Kennard, who said “We had a passionate belief that the collective use of data would lead to the discovery of new knowledge which transcends the results of individual experiments” [2]. As result of this belief, the Cambridge Crystallographic Data Centre (CCDC) was established in 1965, with a remit to collect and share crystal structure determinations of small organic and organometallic molecules, and tabulated knowledge extracted from these. Initially, this sharing was achieved through the printed volumes of molecular structures and dimensions [3, 4]. As these volumes became increasingly unwieldy, electronic computing methods came to the fore, with early software completed by 1978 [5, 6]. This enabled systematic search and analysis, and the systems evolved into the incredibly sophisticated tools we have today [7, 8]. Remaining central to the activities of the Centre is the scientific processing of crystal structure data into a structured database known as the Cambridge Structural Database (CSD).

As the CSD has evolved, so too has the way in which crystal structure data are published. Initially this was as printed tables in journal articles or as supplementary information, both of which needed to be manually retyped. Later, information became available electronically and the advent and adoption by the community of a standard crystallographic information file/framework (CIF) [9] marked a change to almost entirely electronic sharing.

Throughout its near 50 years history, the CCDC has been directed by the objectives enshrined in its Memorandum and Articles of Associations, the formal governing document of the organisation lodged with the UK Charity Commission, the regulator for charities in England and Wales. The CCDC exists for the purpose of advancing chemistry and crystallography for the public benefit through the provision of high quality information services and software. The manner in which this has been achieved has changed dramatically over the years but a key aim has always been to share not just the original datasets but to also make it easy for others to access and apply the knowledge that can be derived from crystallographic data. The aim is to provide timely access to data and knowledge from a range of different contexts and to do so sustainably, so the benefit can be realised by future generations and not just those of today. This article looks at the challenges associated with achieving this.^1^

## Sharing structures

A typical structure determination involves modelling 3D coordinates from processed structure factor data which represents the amplitudes and phases of waves diffracted from a crystal lattice. Structure factors are in turn derived from raw diffraction image data collected from an instrument. The CCDC primarily concerns itself with the modelled 3D coordinates although it has become increasingly common for structure factor data to be included along with the coordinates.

In April 2014, the number of structures in the CSD topped 700,000 [10] with 47,598 structures added in 2013 [11]. The headline number of structures published and entered into the database masks a larger number, mostly hidden from public view. Structures identified or received by the CCDC are typically shared with referees as part of the peer review system: modifications are suggested and revised structures received from authors. The same dataset may also be associated with more than one publication. Structures are often received multiple times and the release of these to the public must be precisely orchestrated to match the publication system. This results in the need for a sophisticated informatics system that can respond to ever increasing numbers of structures.

CCDC has therefore developed an internal informatics system, known as CSD-Xpedite [12]. The CCDC has, historically, always had a need for technological solutions that has run ahead of the standard solutions available. However, commercial solutions for data and transaction management [13] and document management [14] are now available and used in CSD-Xpedite to reduce the problem to one of system configuration (still remarkably complex) rather than ab initio system development. CSD-Xpedite automates many of the steps involved in managing depositions from submission through to publication. It also provides opportunities for integration with publisher workflows so that, for example the CCDC is automatically notified when a paper including a crystal structure has been published.

For the CCDC to achieve its aims of sharing knowledge as well as data, effective management and timely release of deposited datasets is just part of the story. The most crucial aspect of the creation of the CSD is the accurate representation of the ‘chemistry’ of the substance that has been analysed. A deposited CIF usually contains only a minimal representation of the chemistry and rarely includes bond types and charge assignments. These must therefore be deduced from 3D coordinates or by consulting an associated article. Using information in a published article presents many programmatical challenges and requires the input of expert structural chemists (‘Editors’ in the parlance of the CCDC). Automatic deduction of chemical representations purely from 3D coordinates is also a complex task, particularly when one considers that the aim of a crystallographer is often to determine the structure of a hitherto unseen molecule. Even in a world where no errors were made, the challenges presented by crystallographic disorder, polymeric compounds and complex metallo-organic structures are formidable – and we don’t live in an error-free world.

In order to help overcome these scientific challenges, the CCDC has developed a program known as DeCIFer, at the heart of which is an algorithm that attempts to automatically assign chemistry to structures [15]. This uses a Bayesian approach to suggest a likely chemical representation based on a combination of the observed geometry of molecules in a structure and prior assignments captured in CSD entries that have been validated by Editors. DeCIFer also includes algorithms for automatically resolving disorder based on occupancy data in the deposited CIF. This does not automatically overcome all problems but the overall success rate is about 74 %. As the system bases its assignments on the current contents of the CSD, it will naturally improve with time, but of course this improvement is likely to be offset by the new achievements of synthetic chemists. Recognising that 100 % success is therefore likely to remain an unrealistic proposition, all assignments are accompanied by a reliability score which indicates how well the algorithm assesses the assignment to be.

A *modus operandi* has been established whereby an automatic assignment is made immediately a structure is processed and this structure is made available, *caveat emptor*, to the world through the CSD-Xpress facility, along with an indication of the assignment reliability [16]. Structures are then reviewed by Editors, guided by the DeCIFer assignments, before being entered into the CSD itself. The aim of this curation is to ensure that the structure is ready to use by others without the need to spend precious research time on structure correction, and is of appropriate quality from which to generate derived knowledge bases.

## Sharing knowledge

Core to the CSD System are software and services that facilitate lookup of crystal structures [17, 18]. These are fine if the user has a degree of confidence that crystal structure data are available for a compound of interest and they simply want to find it. But what if an individual doesn’t know that crystal structure data might be available and of interest? In this case, services that facilitate access to data and knowledge from other contexts are needed.

### Linking from other resources

Links to structures from scientific publications are, of course, available. Such links are to individual datasets, using CCDC accession IDs (CCDC Number), to all structures associated with a publication or references cited by a publication, enabling discovery across publishers. Scientists following these links will arrive at a landing page that provides free access to the data of record and links to the enriched entries in the CSD. Similarly, non-publication centric resources, such as ChemSpider [19] and PubChem [20], offer the opportunity to provide links to crystal structures. In collaboration with DataCite [21], Digital Object Identifiers are now generated for structures, providing another means of facilitating such links.

One of the most common requirements for a small molecule crystallographer is the ability to check whether a particular sample has been studied before. This can be achieved through a reduced cell search [22] which allows the rapid identification of potentially identical samples as the first step in crystal analysis. Using a system such as CellCheckCSD [23], it is possible initiate these searches using data fresh from the measuring instrument to avoid accidental structure redeterminations.

### Applying knowledge to macromolecular crystallography

Beyond sharing of data, the CCDC is tasked with sharing the knowledge implicit in the collected body of crystal structure data. An example of this is the use of small molecule geometric information [24] in the validation of ligands bound to proteins [25]. A macromolecular crystallographer, who may lack an in depth knowledge of structural chemistry, is alerted if angles and bonds in any ligand are found to fall outside of the norms suggested by knowledge in the CSD. Further benefits of small molecule crystal structures to this community will be achieved as a result of the assignment and sharing of molecules in the CSD that match ligands in the PDB [26].

In situations where no prior structure exists in the CSD, knowledge from related compounds can still be used to derive refinement restraint dictionaries based on the geometry of fragments present in the ligands. One such a service is provided free to the academic community through Global Phasing’s *GRADE* restraint dictionary generator which uses experimental information when possible, complementing this with calculated restraints when needed [27]. Other modelling and refinement packages such as COOT [28] and Phenix [29] can also exploit knowledge extracted from small molecule crystal structures, providing this information at the point it is most useful—when it can help the scientist get a better result from their experiment rather than applying it to validate their results after the event.

### Exploiting knowledge in CCDC tools

Naturally, the CCDC produces tools that take advantage of the knowledge in the CSD in a range of problem domains. The program SuperStar [30] is able to indicate where particular ligand functional groups will most likely interact with residues defining a protein binding site, based on interaction maps derived from small molecule structures. The protein–ligand docking program, GOLD [31], scores the interactions between proteins and ligands based on CSD derived knowledge of interactions, restricts possible ligand conformations to the most likely, based on conformations observed in small molecule structures and uses specific knowledge about ring geometries [32]. Within the program Mercury, the likelihood of particular hydrogen bonding arrangements in small molecule crystals can be predicted based on the propensity of hydrogen bonds in all previous structures [33].

### Access to knowledge through programming interfaces

Whilst CCDC tools have been developed to help address specific problems faced by scientists working on real life problems in industry and academia, no one organisation can expect to anticipate all scenarios where crystal structure data and knowledge are ripe for exploitation. Neither should any organisation have a monopoly on developing tools using this information. With this in mind, the CCDC has developed application programming interfaces (APIs) that provide access to both data and functionality, unconstrained by existing user interfaces. A Python [34] wrapper around CCDC C++ libraries and RESTful Web Services [35] that sit on top of the Python layer provide programmatic access to the full range of search and analysis functionality, regardless of the initial application domain. Importantly they provide a foundation for users and third parties to integrate access to small molecule crystal structure data and knowledge in a range of different systems including modelling packages, pipelining tools and internal workflows.

## Sharing sustainably

Thus far we have drawn little distinction between those CCDC services that are provided free of charge at point of use and those for which a financial contribution is sought.

The first thing to note is that all identified and deposited data, along with services provided to depositors, referees and publishers are provided free of charge. This extends to software provided for validating CIFs [36] and visualising crystal structures [37]. The CCDC thus provides crystallographers with a (to them) free and sustainable channel to share their output with others. The CCDC receives no public funding in direct support of its data curation activities. Whilst this avoids the inherent uncertainties of relying on periodic grant funding, it does mean that the organisation must generate its own income to support its activities.

### Current sustainability model

Instead, the ongoing *maintenance* of the CSD, the data curation activities and the free provision of the structures of record is provided for by contributions made by academic users of the CSD. An advantage of this arrangement is that the resource is inherently sustainable. Whilst it remains of value to academic scientists and whilst those academic scientists continue to be funded, the small financial contributions made will continue. The *development* of the CSD System is made possible through licensing access to the system and associated software to profit-making organisations. These include organisations involved in pharmaceutical and agrochemical research and development, and those involved in materials science. A consequence of this model is that commercial users do not subsidise academic users; this would make the sustainability of the CSD system predicated on the fortunes of industry. A further consequence is that academic users benefit from developments funded by the industrial sector as these are made available to all.

Whilst this model has supported the CCDC for almost 50 years, it does have the consequence that some restrictions are in place on redistribution of the CSD System. Simply put, if all users could share access to the system, only one user might make a financial contribution and the resource would no longer be sustainable. But requiring any financial contribution, regardless of affordability, for value-added services clearly risks discouraging access, particularly by the casual user. It is therefore incumbent on a charity such as the CCDC to identify models that allow these barriers to be lowered or indeed removed.

### Alternative sustainability models

Reviews by Bastow and Leonelli [38], Berman and Cerf [39] point to a number of alternative ways in which data repositories could be funded. Here we consider two alternatives, based on models actively adopted by other data repositories.

The funding model that has served the PDB for over 40 years is to seek public (grant) funding to directly support data curation and access activities. It is a testament to the efforts of PDB staff in raising these funds and the good-faith of funding organisations that this model has sustained the invaluable activities of the PDB over this period. However, this particular funding model is not guaranteed to be sustainable. In recent years resources that were once freely accessible have needed to make elements available via subscription due to lack of stable funding [40] and others see their future under threat [41]. The mismatch between the long-term commitment of preserving research data for future generations and the short-term episodic funding typically provided to support only the establishment of such activities is a concern shared by directly funded repositories across a range of disciplines [42].

Dryad [43], a general-purpose data repository for a wide diversity of types of data, was initially established through grant funding with the requirement that it establish an income stream that would make it self-sustaining [44]. The model they chose was one of charging researchers to deposit [45]. A concern expressed from some in the wider community soon after this charging model came into effect is that upfront fees such as these will discourage researchers from sharing data in the first place [46].

Given the concerns and pitfalls associated with these examples it is perhaps inappropriate to make significant change to a model of demonstrated sustainability until there are clear signs of an appetite and willingness by researchers to pay to deposit or until there is sufficient confidence that public funds will sustain repositories. Any decisions taken must be sympathetic to the long term duty of care to preserve the research output of the crystallographic community. However, the CCDC should look at ways in which it can provide greater value to the scientific community with the fewest restrictions.

### Easing the burden

As discussed above, although access to individual structures and many other services offered by the CCDC is free, the organisation does seek contributions from users of the CSD. It is, therefore necessary to establish a financial and legal relationship with users. One way of alleviating the burden on the individual researcher is by engaging with centrally-funded initiatives aimed at providing access across a region. Examples include the EPSRC-funded Chemical Database Service [47, 48] which provides CSD System access to all UK academic institutions and the availability of the CSD System to institutions in Brazil through the Coordenação de Aperfeiçoamento de Pessoal de Nível Superior (CAPES) [49]. Access to the CSD System in other countries is often provided through a network of National Affiliated Centres, who not only take on the burden of distributing the CSD System, but often secure funding at the national level, from government sources, or by institutions ‘clubbing together’. Of course, in some regions funding for crystallography is scarce. In these cases the CCDC significantly subsidises the cost of access and ensures that no individual is denied access to data because of a genuine lack of funds.

## Accessibility versus quality

More troublesome than financial barriers are restrictions on reuse of data, put in place to protect both the sustainability of the CSD and to honour the CCDCs responsibilities to the community as custodians of their data.

A desire within the wider scientific community for open access to data that is free of any restrictions, whether financial or otherwise, has led to the creation of collections of CIF files [50, 51]. The Crystallography Open Database for example [50] hosts CIFs for inorganic structures as well as small molecule organics and metal-organics, donated or downloaded from publisher web sites. At the time of writing this contained 265,575 entries [52] whilst the CSD and the ICSD combined contained 880,880 entries.^2^ The impact of this difference in coverage at a practical level was highlighted in a recent study that compared the use of data from CSD and COD in predicting 3D structure conformations [53]. This showed that the number of unique substructure fragments derived from the COD was just 9 % of those that could be derived from the CSD. Moreover, the curation steps needed to prepare structures from the COD for this study included identification of errors such as non-standard representations, partially specified structures and missing atoms, missing bonds and hydrogens. These represent a few of the steps undertaken by the CCDC as part of the curation processes applied to structures in the CSD. If every researcher had to repeat these steps then this represents a significant investment of time and energy that could otherwise be spent on more innovative research.

The investment currently made by the community through financial contributions helps ensure that the Cambridge Structural Database is comprehensive and that structures are fit for use without the need for additional curation. With government and funder policies understandably pushing for greater accessibility to research data we anticipate that finding the right balance between accessibility and quality, whilst being able to continue activities on a sustainable basis will be a challenge for repositories across all disciplines in the years ahead.

## Future prospects

The technique of X-ray crystallography is over 100 years old [54] and in 2014 we celebrate the International Year of Crystallography [55]; the CCDC itself will be 50 years old in 2015. But this pedigree does not mean that there are no more challenges and opportunities surrounding the science of experimental 3D structure determination and the dissemination of data arising from this.

### New types of experimental data

One of the current criteria for entering a structure in the CSD is that it has it has been studied using either X-ray or neutron diffraction, but it is also possible to study compounds using electron diffraction [56]. Recently, Baias et al. [57] have determined the crystal structure of a large drug molecule using a combination of solid state 1H NMR spectroscopy and computational calculations. Then there are crystal forms that have been hypothesised purely computationally using a combination of algorithmic, energetic and knowledge-based techniques [58]. An obvious question then is how far the CSD should move beyond its current content and incorporate data arising from a wider range of analytical techniques.

### Additional experimental data

As noted earlier, the data typically used in the CSD are the coordinates of the final refined model. However, the value of data in the form of structure factors is now appreciated in the small molecule community as it has been for macromolecular crystallographers. Cases of fraud [59] and disputes about the validity of scientific claims [60] have further highlighted the value in crystallographers also depositing structure factors. In line with IUCr recommendations on publication standards for crystal structures [61], the CCDC has accepted structure factors since 2011. These are required by the IUCr’s own journals and we expect to see other journals make these a requirement. A challenge here is making sure that such additional requirements do not impose barriers that discourage authors from publishing in journals with more stringent requirements for deposition of data, a valid if somewhat dispiriting concern raised in discussion of revisions to the Public Library of Science’s Data Policy [62]. The raw data from which structure factor data themselves are derived could also be stored. In contemplating this, economic as well as social factors need to be considered [63] alongside scientific value [64].

### Unpublished structures

A significant challenge for the wider community relates to dissemination of structures that have been determined but never published. The results from a joint IUCr-ICSTI survey of crystallographers undertaken in 2004 revealed more respondents with over 500 *unpublished* structures than there were with more than 500 *published* datasets [65]. Previously unpublished data, or “Private Communications” accounted for 1.3 % of structures in the CSD at the end of 2013. Whilst this may seem small, it would rank at 21 in the list of 111 journals contributing more than 500 structures to the CSD [66]. This, however, is likely to be just the tip of an iceberg, the melting of which will require mechanisms that minimise technical barriers to sharing and promote the value of so doing.

The eCrystals platform [67] developed by the UK National Crystallography Service [68] provides an exemplar of a platform that can help reduce technical barriers. This aims to capture data as an experiment is undertaken and subsequently makes it easy to share these data. Datasets published this way are also harvested by the CCDC and included in the CSD. The value to the researcher can be enhanced by making sure datasets are recognised as legitimate citable objects worthy of the same type of recognition currently afforded to article citations, a tenet that is at the core of recently published principles regarding citation of data [69]. The assignment of DOIs to datasets go a long way to satisfying elements of these principles and offers a value that may incentivise a researcher to invest the extra effort required to make available data that they would not otherwise publish.

We must recognise that there are some structures for which data are less likely to be publically shared. Structures determined by the pharmaceutical, agrochemical and other chemical industries are, understandably, often guarded, as the compounds studied represent potential intellectual property. The CCDC therefore provides these industries with tools that enable them to analyse their compounds alongside the CSD. In addition, it may be possible to facilitate the sharing of the knowledge implicit in these structures by, for example, tapping into the spirit of open innovation currently pervading the pharmaceutical sector [70].

### Storage requirements

The modelled 3D coordinates of a single crystal structure are captured in files of around 20-100kB. The current collection of these files, with their revisions, associated correspondence, derived CSD entries and other associated files currently requires 58 GB of storage. The processed data from which these are derived, the structure factor amplitudes, can be stored in about 500kB for each structure. Although only a small percentage of current datasets include structure factor data (around 1.5 %), we expect this percentage to approach 100 % for newly deposited datasets. This will result in a system requiring around 1 MB of storage per structure for newly deposited datasets, giving a total size of perhaps 500 GB in 2020, which is not likely to present insurmountable challenges for storage or searching. Only if the raw data output from instruments is archived would the fundamental architecture of the system need to change, as such data can easily exceed 500 MB per experiment.

## Final remarks

In a different world, data would be streaming off instruments straight into a public repository, regardless of a scientist’s intention to publish. Chemistry would be automatically and reliably assigned with no need for manual validation and the resulting structures made freely accessible for any purpose to the world and its machines. Automated processes would ensure that there were always links to data from relevant resources whether established or new. The repository would be supported by an infinite storage cloud that discriminated not on size of dataset. And, where costs were incurred, there would, perhaps, be a pot of gold on hand at the end of a rainbow.

Of course this utopian vision is not a reality yet, particularly where the pot of gold is concerned and data repositories must be creative in identifying sources of funding to sustain their activities for the long term benefit of the scientific community. In so doing they must also make tough choices about the levels of quality, accessibility, comprehensiveness and longevity that best satisfy the needs of the communities they serve. Happily though, there are many elements of this world in place. Systems that lower technical barriers to the deposition of data and join up with publication workflows are in place. Automatic assignment of chemistry can be achieved and although not perfect, this can alert us to situations where the assignment may be unreliable. All structures of record are freely available and mechanisms are in place to ensure these are discoverable from other resources. Interoperability between systems is being made easier with the adoption of standard identifiers such as DOIs.

Most excitingly, data sharing has become a topic of great interest and discussion within the wider community. This has brought to the fore challenges and opportunities of specialist data repositories and, with this increased community engagement, we all look set to continue to benefit from the tremendous achievements in crystallography.

## Footnotes

1 In parallel to the CSD, systems also evolved to provide access to crystallographic data of other molecules, for example inorganics, through the inorganic crystal structure database (ICSD) [71] and CrystMet [72], and macromolecules through the protein data bank (PDB) [73]. As the focus of the CCDC remains on small organic and metal-organics, subsequent discussions will focus on this area.

2 As at 10 March 2014, the advertised number of structures in the ICSD was 166,842 [74]. The number of structures available through WebCSD was 714,038; this included 19,168 CSD X-Press entries.
